# Observational Study of the Association of First Insulin Type in Uncontrolled Type 2 Diabetes with Macrovascular and Microvascular Disease

**DOI:** 10.1371/journal.pone.0049908

**Published:** 2012-11-14

**Authors:** Gillian C. Hall, Alex D. McMahon, Dawn Carroll, Philip D. Home

**Affiliations:** 1 Grimsdyke House, London, United Kingdom; 2 Reader in Epidemiology, University of Glasgow Dental School, Glasgow, United Kingdom; 3 Sanofi-aventis Ltd, Guildford, Surrey, United Kingdom; 4 Newcastle University, Newcastle upon Tyne, United Kingdom; Thomas Jefferson University, United States of America

## Abstract

**Aims:**

To compare the risk of vascular disease, HbA1c and weight change, between first prescribed insulins in people with type 2 diabetes.

**Methods:**

People included in THIN United Kingdom primary care record database who began insulin (2000–2007) after poor control on oral glucose-lowering agents (OGLD) were grouped by the number of OGLDs in their treatment regimen immediately before starting insulin (n = 3,485). Within OGLD group, Cox regression compared macrovascular (all-cause mortality, myocardial infarction, acute coronary syndrome and stroke) and microvascular disease (peripheral neuropathy, nephropathy, and retinopathy) between insulin type (basal, pre-mix or Neutral Protamine Hagedorn, NPH) while ANCOVAs compared haemoglobin A_1c_ (HbA_1c_) and weight change.

**Results:**

Mean follow-up was 3.6 years. Rates of incident macrovascular events were similar when basal insulin was compared to pre-mix or NPH, adjusted hazard ratio versus basal: pre-mix 1.08 (95% CI 0.73, 1.59); NPH 1.00 (0.63, 1.58) after two OGLDs, and pre-mix 0.97 (0.46, 2.02); NPH 0.77 (0.32, 1.86) after three OGLDs. An increased risk of microvascular disease in NPH versus basal after 3 OGLDs, adjusted hazard ratio1.87 (1.04, 3.36), was not seen after two agents or in comparisons of basal and pre-mix. At one year, after two OGLDs, weight increase was less with basal compared with pre-mix. After three OGLDs, mean HbA_1c_ had reduced less in basal versus pre-mix or NPH at 6–8 and at 9–11 months, and versus pre-mix at 12–14 months.

**Conclusion:**

We found no difference in the risk of macrovascular events between first insulins in the medium term when started during poor glycaemia control. The increased risk of microvascular events with NPH warrants further study. In certain groups, first use of basal insulin was associated with less gain in weight and decrease in HbA_1c_ compared to other insulins.

## Introduction

A short-term aim of type 2 diabetes management is good glycaemic control with a longer-term objective of reducing complications. People with known type 2 diabetes have a two- to four-fold increased risk of ischaemic heart disease (IHD) and an increased risk of microvascular disease [1]. Initial pharmaceutical treatment is usually with an oral therapy. However, a steady decline in islet β-cell function usually results in progressive hyperglycaemia so a stepwise escalation in the number of OGLDs prescribed is frequently required to maintain glycaemic control. Eventually, many people require insulin to maintain glycaemic control as insulin secretion decreases [2]. The choice when beginning insulin treatment is usually between human NPH insulin (NPH) injected once or twice daily, a long-acting basal insulin analogue (basal) or a pre-mix preparation. Current United Kingdom (UK) National Institute for Health and Clinical Excellence guidance recommends beginning with NPH but to consider long-acting analogues or pre-mixed insulin formulations under specified circumstances [3].

There is little published data from routine care as to the effect of the choice of insulin on HbA_1c_ levels, body weight change and subsequent vascular disease. Clinical trials in type 2 diabetes have reported less weight increase and less reduction in HbA_1c_ but fewer hypoglycaemic episodes during treatment with basal insulin [4], [5]. No evidence of a greater risk of the development or progression of diabetic retinopathy between treatments after five years was reported in a trial of insulin glargine versus NPH insulin [6]. The ORIGIN study reported that over 6 years, insulin glargine did not increase the risk of cardiovascular outcomes compared to standard care in those with cardiovascular risk factors plus impaired fasting glucose, impaired glucose tolerance, or type 2 diabetes [7]. The aim of the present study was to investigate whether, in routine clinical practice, the occurrence of vascular outcomes, and the change in HbA_1c_ and body weight, was different depending on which insulin type was prescribed first.

## Methods

### Ethics Statement

The manuscript describes a non-interventional study so informed consent was not required. The protocol was approved by the Cambridgeshire 4 Research Ethics Committee (UK).

### Source Population

All data came from The Health Information Network (THIN), an observational database containing information collected in computerized primary care practices throughout the UK (http://www.thin-uk.com/) [8]. Demographic and administrative data, primary care diagnoses and prescription treatments are routinely recorded against date in individual patient records. Details of referrals, secondary care diagnoses and deaths are also captured due to the structure of the UK health service. The UK has a publicly funded healthcare system which is free to UK residents. Within this system patients are registered with one general practitioner who is involved in primary healthcare, specialist referrals, and hospital admissions. Consequently the electronic primary care record is a centralised source of information on these events. Major health events from before computerization are added retrospectively. Data on preventive medicine are recorded including details of any annual diabetes review. Medical events are automatically coded at entry using the Read coding system [9]. The source population comprised 202 456 people with a record of diabetes from 381 practices which received laboratory results electronically.

### Cohort Formation and Treatment Definition

People (n = 105 845) identified were those who had received at least one prescription for an OGLD after 1^st^ January 2000 but had no prior or current prescription for insulin. A sub-group, who began insulin treatment between 1^st^ January 2000 and the most recent data collection (up to 30^th^ November 2008), were selected. Beginning insulin was defined as more than one insulin prescription as short-term insulin is commonly used during periods of ill health and we were interested in the effects of long-term treatment. In that population, the duration of each OGLD type (metformin, gliclazide, pioglitazone, etc.) was calculated from 1^st^ January 2000 up to beginning insulin. The number of OGLD types still prescribed immediately prior to starting insulin (baseline) was counted. It was assumed that a treatment stopped as a new drug was started if there were no further prescriptions for the first OGLD within a time period which was double that of the duration of the last prescription for this drug. OGLD duration was calculated from the number of items and dosage prescribed. When insufficient dosage information was recorded for the calculation of duration the most common dose for that form and strength of the OGLD was used.

Those who started insulin were initially grouped into nine cohorts depending on the combination of the number of baseline therapies (one, two or three oral agents) and whether basal, NPH or pre-mix was started. The number of baseline therapies was used in the groupings as the stage at which insulin is started can be related to diabetes duration, severity and concomitant disease. Patients initiating other insulins or combinations of insulins and those restarting insulin therapy were not included. Consequently analyses were always completed for mutually exclusive groups. The date of the first insulin prescription was taken as the index date. People with more than one year of electronic record pre- or post-index date, aged between 18 and 80 were included. The one year pre-insulin period gave sufficient medical record length to identify new insulin users and those with a history of the study outcomes. People with no interpretable HbA_1c_ record in the three months prior to index date, the most recent HbA_1c_ <7.5% (58 mmol/mol) or no body weight record in the previous 15 months, were excluded. The HbA_1c_ criteria were chosen to ensure that only people in poor blood glucose control were included. Comparisons of those who started insulin from one baseline OGLD are not reported. This was because commencing insulin after one agent was uncommon with few vascular events reported and a parallel study comparing OGLD and insulin found that patients who initiated insulin treatment from a regimen of one oral therapy differed from other patients in terms of both medical history and data recording [10].

### Outcomes

The primary cardiovascular endpoint was a composite of all-cause mortality, myocardial infarction, acute coronary syndrome, or stroke. Angina and coronary procedures were added to the primary cardiovascular endpoint in sensitivity analyses. The microvascular endpoint was a composite of peripheral neuropathy, diabetic nephropathy, and retinopathy. The first event was taken for the composite endpoints. Patients with events prior to the index date were excluded from the relevant analysis. Amputations were identified but in insufficient numbers to perform comparative statistical analysis. Change in HbA_1c_ and body weight in the year after index date were secondary outcomes.

### Baseline Covariates

The following potential confounding baseline variables were extracted: age, sex, recorded duration of diabetes, most recent HbA_1c_, year of starting insulin, OGLD and cardiovascular therapies, history of vascular disease and its risk factors. OGLD (sulfonylurea, metformin, thiazolidinediones, combinations of these or other) and cardiovascular therapies (British National Formulary categories 2.2–2.6 [11]) were defined as a prescription in the 16 weeks up to and including the index date. History of vascular disease included IHD (MI, angina, coronary revascularisation, or IHD), cerebrovascular disease (any stroke, transient ischaemic attack or cerebral insufficiency/ischaemia), peripheral arterial disease, heart failure, hypertension, or microvascular complications (as above). Other cardiovascular risk factors ascertained included: hyperlipidaemia (as a diagnosis, cholesterol: high density lipoprotein ratio >4.5 or total serum cholesterol >5.2 mmol/l); body mass index (BMI); smoking status; estimated glomerular filtration rate in the previous year [12], [13], microalbuminuria defined as a diagnosis or an abnormal urine albumin concentration or albumin: creatinine ratio.

### Analyses

The baseline characteristics of the total cohorts (before exclusions for prevalent disease) were described. Comparisons were made for basal versus NPH and basal versus pre-mix with the same number of baseline OGLDs. Follow-up started on the date of the first insulin prescription plus 12 months and was censored on the date of the first outcome for that analysis, transfer out of the practice or last data collection. Outcomes in the first 12 months of treatment were not included in the analysis as the insulin was unlikely to have an effect on vascular outcomes in a short time scale. All events from the year after insulin was started were included, regardless of further treatment changes or inclusion in additional cohorts, in a similar manner to intention to treat analysis in randomised clinical trials. This design was chosen because of the time for the effects of poor glycaemic control on vascular outcomes to become evident and because censoring when a patient joined a second cohort could have introduced bias with shorter follow-up in patients with worse control.

Adjusted hazard ratios of the association between our treatment categories and incident (first event) microvascular and macrovascular outcomes were compared using Cox regression models. People with a pre-insulin history of any disease within that composite outcome, or with outcomes in the first year or who transferred out during this time, did not contribute to that analysis. Each baseline covariate was included as a variable in the model for the primary macrovascular outcomes with the microvascular conditions included separately and year of first insulin treated as a continuous variable. Angina and coronary procedures were removed as covariates when these were added to the composite outcomes in sensitivity analyses. All baseline variables except microvascular disease itself were included in the microvascular disease models.

The association of treatment category with body weight change and HbA_1c_ in the year after insulin initiation were investigated with the treatment groups compared as outlined above adjusting for baseline age, sex, duration of diabetes, year of escalation, BMI, HbA_1c_, and oral treatment regimen. The changes in weight from baseline to approximately one year (10–15 months) and HbA_1c_ over three month intervals (4–14 months) were compared using ANCOVA least squares means. All data analysis was performed using SAS software (version 9.1, Cary, NC, USA).

## Results

There were 8149 people who started insulin after two or three OGLDs of whom 2432 had less than one year history or follow-up; 3043 had no interpretable baseline HbA_1c_ record over 7.5% (58 mmol/mol) available in the 90 days before insulin was started; 558 had no baseline weight reading and 623 were <18 or >80 years of age. After all exclusions, 2537 people began insulin after two OGLDs and 890 after three. NPH was the least common first insulin being prescribed to less than 20% of people in any group (Table 1).The mean total follow-up period was 3.6 years across groups. Baseline therapies were similar across insulin types with the majority of people on two baseline therapies being prescribed metformin and a sulfonylurea with a thiazolidinedione added as the third agent (Table 2).

**Table 1:** Characteristics of the insulin cohorts.

	2 oral baseline treatments				3 oral baseline treatments		
	basal analogue	pre-mix	NPH	basal analogue		pre-mix	NPH
Total (*n*)	1033	1063	441	394		336	160
Age: (yr) mean (SD)	61.6 (11.1)	61.1 (11.1)**	61.8 (11.1)	61.0 (10.2)		61.7 (9.9)**	63.9 (10.2)**
Total follow-up (yr) mean (SD)	3.1 (1.2)	4.1 (1.9)**	4.1 (1.8)**	2.7 (1.2)		3.7 (1.8)**	4.0 (1.9)**
Diabetes duration (yr) mean (SD)	7.8 (5.1)	8.4 (6.7)*	7.9 (4.9)	9.6 (6.3)		9.1 (5.8)	9.4 (5.3)
Female *n* (%)	458 (44.3)	441 (41.5)	207 (46.9)	147 (37.3)		127 (37.8)	62 (44.3)
Escalation 2004–2007 n (%)	924 (89.5)	579 (54.5)**	233 (52.8)**	361 (91.6)		217 (64.6)**	83 (59.3)**
Cholesterol (mmol/l) mean (SD)	6.3 (1.6)	6.3 (1.5)	6.1 (1.7)*	6.4 (1.6)		6.5 (2.9)	6.3 (1.3)
BMI (kg/m^2^) mean (SD)	31.1 (6.4)	30.0 (6.0)**	30.9 (6.8)	31.5 (6.9)		30.3 (6.4)*	30.4 (5.5)
Smoker *n* (%)	186 (18.0)	194 (18.3)	84 (19.1)	59 (15.0)		61 (18.2)	21 (15.0)
eGFR (ml/min/1.73 m^2^) n (%):							
<30	8 (0.8)	17 (1.7)*	3 (0.7)	3 (0.8)		5 (1.6)	1 (0.7)
30–60	220 (21.6)	253 (24.7)	78 (18.3)	73 (18.9)		69 (21.6)	33 (24.4)
History of (*n* (%):							
Macrovascular disease^a^	142 (13.8)	173 (16.3)	66 (15.0)	48 (12.2)		48 (14.3)	17 (12.1)
Microvascular disease^b^	251 (24.3)	281 (26.4)	113 (25.6)	122 (31.0)		110 (32.7)	35 (25.0)
Cardiovascular drug use	957 (92.6)	904 (85.0)**	384 (87.1)**	378 (95.9)		306 (91.1)**	124 (88.6)**
Coronary revascularisation	71 (6.9)	69 (6.5)	29 (6.6)	29 (7.4)		23 (6.9)	10 (7.1)
Heart failure	52 (5.0)	72 (6.8)	26 (5.9)	16 (4.1)		23 (6.9)	6 (4.3)
Angina	207 (20.0)	227 (21.4)	93 (21.1)	69 (17.5)		67 (19.9)	23 (16.4)
Cerebral insufficiency	50 (4.8)	45 (4.2)	15 (3.4)	11 (2.8)		19 (5.7)	6 (4.3)
Hypertension	579 (56.1)	613 (57.7)	236 (53.5)	224 (56.9)		187 (55.7)	76 (54.3)
PAD	71 (6.9)	78 (7.3)	24 (5.4)	22 (5.6)		21 (6.3)	5 (3.6)
Amputation	10 (1.0)	10 (0.9)	5 (1.1)	2 (0.5)		8 (2.4)	1 (0.7)
Microalbuminuria	102 (9.9)	85 (8.0)	42 (9.5)	55 (14.0)		32 (9.5)	14 (10.0)
Hyperlipidaemia	691 (66.9)	682 (64.2)	282 (64.0)	283 (71.8)		225 (67.0)	92 (65.7)

eGFR estimated glomerular filtration rate, PAD peripheral arterial disease.

a Composite of MI, acute coronary syndrome or stroke;

b Composite of neuropathy, nephropathy, and retinopathy;

* p<0.05

** p<0.01, compared to basal insulin, chi-squared or t-test for means.

**Table 2:** Glucose-lowering therapy prescribed in the 112 days before starting insulin by insulin type and number of baseline oral therapies^a^ (*n* (% of cohort)).

	2 oral baseline treatments			3 oral baseline treatments		
	basal analogue	pre-mix	NPH	basal analogue	pre-mix	NPH
Sulfonylurea	4 (0.4)	5 (0.5)	3 (0.7)	0 (0.0)	0 (0.0)	0 (0.0)
Metformin+sulfonylurea	685 (66.3)	668 (62.8)	321 (72.8)	12 (3.0)	15 (4.5)	14 (8.8)
Metformin+thiazolidinedione	143 (13.8)	135 (12.7)	39 (8.8)	0 (0.0)	0 (0.0)	0 (0.0)
Metformin+ other	15 (1.5)	18 (1.7)	12 (2.7)	2 (0.5)	1 (0.3)	0 (0.0)
Sulfonylurea+thiazolidinedione	120 (11.6)	166 (15.6)	42 (9.5)	1 (0.3)	6 (1.8)	2 (1.3)
Sulfonylurea+other	9 (0.9)	13 (1.2)	3 (0.7)	0 (0.0)	0 (0.0)	0 (0.0)
Thiazolidinedione+other	2 (0.2)	2 (0.2)	0 (0.0)	0 (0.0)	0 (0.0)	0 (0.0)
Metformin+sulfonylurea+thiazolidinedione	47 (4.5)	37 (3.5)	14 (3.2)	309 (78.4)	239 (71.1)	83 (51.9)
Metformin+thiazolidinedione+other	4 (0.4)	4 (0.4)	0 (0.0)	17 (4.3)	6 (1.8)	5 (3.1)
Sulfonylurea+thiazolidinediones+other	1 (0.1)	2 (0.2)	0 (0.0)	12 (3.0)	6 (1.8)	1 (0.6)
Metformin+sulfonylurea+other	2 (0.2)	11 (1.0)	7 (1.6)	39 (9.9)	55 (16.4)	32 (20.0)
All groups	1 (0.1)	2 (0.2)	0 (0.0)	2 (0.5)	8 (2.4)	3 (1.9)

a The counts include the OGLDs prescribed in the 112 days to index date, so the total number of baseline therapies may be greater than the number the people were taking at baseline. The drug regimen at index date may have included more than one drug in the same category.

The risk of microvascular disease was greater in the NPH group compared to basal analogue insulin after three oral agents, but not after two OGLDs, adjusted hazard ratios 1.87 (95% confidence interval 1.04, 3.36) and 1.03 (0.73, 1.45) respectively (Table 3). No difference in microvascular events was seen between basal and pre-mix after either two or three OGLDs. No statistically significant difference in the rate of macrovascular events, or macrovascular events plus angina or coronary revascularisation were seen after adjustment for baseline characteristics (Table 3). However, the adjusted hazard ratio for the comparison of macrovascular disease plus angina in basal analogue versus premix after two OGLDs was 1.50 with a lower confidence interval of 0.99.

**Table 3:** Comparison of incident macro- and micro-vascular disease between insulin treatment groups.

	2 oral baseline treatments				3 oral baseline treatments			
Endpoint		basalanalogue	pre-mix	NPH	basal analogue	pre-mix		NPH
Macrovascular^a^								
* n* (%)		51 (5.8)	117 (13.3)	42 (11.3)	19 (5.5)	26 (9.4)		13 (10.9)
Unadjusted HR (95% CI)		Reference	1.41 (1.00 1.98)	1.18 (0.78 1.79)	Reference	1.16 (0.64 2.11)		1.11 (0.54 2.29)
Adjusted HR (95% CI) b			1.08 (0.73 1.59)	1.00 (0.63 1.58)		0.97 (0.46 2.02)		0.77 (0.32 1.86)
Microvascular^c^								
* n* (%)		100 (13.9)	133 (18.4)	68 (22.2)	33 (13.2)	42 (19.5)		31 (32.3)
Unadjusted HR (95% CI)		Reference	0.93 (0.71 1.21)	1.12 (0.82 1.53)	Reference	1.02 (0.64 1.62)		*1.73 (1.05 2.84)*
Adjusted HR (95% CI)^b^			0.79 (0.58 1.07)	1.03 (0.73 1.45)		1.05 (0.62 1.80)		*1.87 (1.04 3.36)*
**Sensitivity analyses**								
Macrovascular + Angina								
* n* (%)		42 (5.5)	123 (16.0)	39 (12.1)	23 (7.5)		28 (11.5)	13 (12.3)
Unadjusted HR (95% CI)		Reference	*1.93 (1.35 2.76)*	1.41 (0.91 2.20)	Reference		1.03 (0.59 1.80)	0.94 (0.47 1.89)
Adjusted HR (95% CI)^b^			1.49 (0.99 2.25)	1.23 (0.76 1.99)			0.76 (0.39 1.51)	0.75 (0.33 1.70)
Macrovascular+coronary revascularisation								
* n* (%)		50 (5.8)	129 (15.2)	42 (11.6)	18 (5.4)		27 (10.0)	14 (12.3)
Unadjusted HR (95% CI)		Reference	*1.62 (1.16 2.27)*	1.21 (0.80 1.83)	Reference		1.33 (0.72 2.43)	1.41 (0.69 2.87)
Adjusted HR (95% CI)^b^			1.31 (0.89 1.91)	1.01 (0.64 1.59)			1.30 (0.63 2.68)	1.32 (0.56 3.14)

a Composite of all-cause mortality, MI, acute coronary syndrome or stroke.

b Covariates are described in the Methods text.

c Composite of neuropathy, nephropathy, and retinopathy.

In all cohorts and at all time periods there was a decrease in HbA_1c_ after beginning insulin but the mean HbA_1c_ remained above the target of 7.0–7.5% (53–58 mmol/mol) in all groups (Table 4). There was no difference in HbA_1c_ change between any groups who began insulin after two oral agents. In those who had three baseline therapies there was a smaller decrease in HbA_1c_ when basal analogue was begun compared to both pre-mix (6 to 14 months after index date) and NPH (6 to 11 months after index date).

**Table 4:** Baseline and change from baseline in HbA_1c_ and body weight and comparison^a^ of change between the insulin cohorts.

	2 oral baseline treatments					3 oral baseline treatments					
	Basal mean (*n)*	pre-mixmean (*n)*	NPHmean (*n)*	basal v pre-mix unadjusted/adjusted^a^	basal v NPH unadjusted/adjusted^a^	Basalmean (*n)*	pre-mix mean (*n)*	NPHmean (*n)*		basal v pre-mix unadjusted/adjusted^a^	basal v NPH unadjusted/adjusted^a^
**HbA_1c_ (%)^C^**											
Baseline	9.7 (1033)	10.2 (1063)	9.9 (441)	*<0.01* b	*<0.05* b	9.6 (394)	10.2 (336)		9.8 (140)	*<0.01* b	ns b
Change from baseline											
3–5 months	−1.3 (690)	−1.6 (620)	−1.5 (276)	−0.01 (−0.15, 0.12)	−0.06 (−0.23, 0.11)	−0.8 (263)	−1.3 (190)		−1.1 (79)	0.00 (−0.27, 0.26)	−0.14 (−0.49, 0.21)
				−0.06 (−0.20, 0.08)	−0.13 (−0.30, 0.05)					0.03 (−0.24, 0.30)	−0.06 (−0.41, 0.30)
6–8 months	−1.4 (608)	−1.7 (550)	−1.5 (253)	−0.04 (−0.19, 0.12)	0.07 (−0.12, 0.26)	−0.8 (220)	−1.6 (180)		−1.5 (89)	−*0.39 (*−*0.66,* −*0.12)*	−*0.61 (*−*0.95,* −*0.28*
				−0.06 (−0.22, 0.10)	0.04 (−0.16, 0.24)					−*0.31 (*−*0.59,* −*0.03)*	−*0.44 (*−*0.79,* −*0.09)*
9–11 months	−1.5 (556)	−1.8 (529)	−1.6 (234)	0.00 (−0.16, 0.16)	0.10 (−0.10, 0.31)	−0.8 (215)	−1.9 (170)		−1.6 (78)	−*0.56 (*−*0.85,* −*0.27)*	−*0.64 (*−*1.01,* −*0.27)*
				−0.03 (−0.20, 0.14)	0.13 (−0.09, 0.34)					−*0.50 (*−*0.80,* −*0.20)*	−*0.53 (*−*0.92,* −*0.14)*
12–14 months	−1.4 (567)	−1.7 (531)	−1.6 (229)	0.00 (−0.16, 0.16)	−0.10 (−0.30, 0.11)	−1.0 (224)	−1.7 (169)		−1.5 (77)	−*0.34 (*−*0.60,* −*0.08)*	−0.31 (−0.64, 0.02)
				0.00 (−0.17, 0.17)	−0.04 (−0.25, 0.17)					−*0.29 (*−*0.55,* −*0.02)*	−0.16 (−0.50, 0.19)
**Body weight (kg)**											
Baseline	88.6 (1028)	85.7 (1057)	87.6 (429)	*<0.01* b	ns^b^	89.9 (392)	87.2 (331)		86.5 (137)	ns^b^	ns^b^
Change from baseline											
10–15 months	1.7 (634)	4.0 (679)	2.8 (258)	*−2.22 (−2.82, −1.62)*	*−1.61 (−2.24, −0.98)*	1.9 (267)	4.0 (228)		3.2 (85)	*−1.96 (−3.04, −0.88)*	*−*1.27 (*−*2.76, 0.21)
				*−0.97 (−1.78, −0.17)*	*−*0.70 (*−*1.52, 0.11)					*−*0.94 (*−*2.03, 0.16)	*−*0.53 (−2.03, 0.97)

a ANCOVA mean difference (95% CI); adjusted for age, sex, duration of diabetes, year of escalation, baseline BMI, and HbA_1c_ and oral treatment.

b t-test.

c Conversion to mmol/mol (% units −2.15) ÷0.0915 for absolute values, (% unit ÷ 0.0915) for change in values. NS, non-significant.

Body weight increased after commencing insulin in all cohorts (Table 4). The increase in weight was least in the basal analogue cohorts, but after adjustment for baseline variables the difference was significant only when the basal treatment group was compared to pre-mix in those who had been on two oral agents.

## Discussion

Our data suggest that, in routine practice, people with type 2 diabetes and initially poor glycaemic control have a similar risk of major macrovascular events (MI, acute coronary syndrome, stroke or death) in the medium term regardless of what type of insulin is started. The mean follow-up was 3.6 years although events in the first year were not included. Fewer patients started insulin after three oral agents resulting in a small number of outcomes and upper 95% confidence intervals close to two. The sensitivity analysis comparing macrovascular disease plus angina in premix insulin versus basal analogue after two OGLDs had a hazard ratio of 1.49 and a lower 95% confidence interval of 0.99 although the same comparison after three oral agents did not show a difference in risk between the insulins with a hazard ratio of 0.76 (95% confidence interval 0.39, 1.51).

Rates of incident microvascular conditions were similar in the treatment groups except in the comparison between basal insulin and NPH after a regimen of three oral agents. The increased rate after NPH is unlikely to be due to better short-term glycaemic control as there was no difference in baseline HbA_1c_ levels but a statistically significant greater improvement in glycaemic control from 6 to 11 months after starting NPH rather than basal insulin. A sharp increase in both testing for diabetes complications [14] and diagnosis of some conditions has been reported after the introduction of the 2004 UK Quality and Outcomes Framework which provided incentives for routine surveillance of people with diabetes. An increased microvascular risk could be due to NPH being started before 2004 more frequently than was the new basal insulin. However, inclusion of year of first insulin in the adjusted analysis should have accounted for this difference. While no difference in the rate of microvascular disease was found after escalation from the larger number of patients who started insulin after two oral agents, there were anticipated differences between those who started insulin from two rather than three oral agents, such as similar HbA_1c_ after a shorter duration of diabetes, which might impact on the development of these complications. This finding therefore warrants further investigation. Differences in weight gain and glycaemic control were identified between insulin groups in the first year of treatment. Starting basal insulin was associated with less reduction in HbA_1c_ than other insulins after three oral agents but less weight gain than pre-mix after two oral agents.

No observational studies or clinical trials were found which specifically aimed to compare the rate of macrovascular disease between first insulins. The Treating to Target in Type 2 diabetes (4-T) clinical trial found no significant difference in death rate between basal, biphasic and prandial insulin regimens over 3 years, but a higher rate of death from cardiovascular disease in the prandial than the biphasic and basal groups [15]. A five-year trial of insulin glargine versus NPH insulin treatment in patients with type 2 diabetes mellitus showed no evidence of a greater risk of the development or progression of diabetic retinopathy between treatments [6]. A smaller decrease in HbA_1c_ in basal insulin versus NPH treated patients was reported in that trial and in the 4-T study when basal insulin (detemir) was compared to both biphasic and prandial treatment [4]. HbA_1c_ levels later converged in the 4-T study after usual addition of other insulins, [15] and in the Treat to Target study more glargine users eventually achieved a target of HbA_1c_ ≤7.0% (53 mmol/mol) without nocturnal hypoglycaemia [16].

Less weight gain in the basal group in routine care was consistent with several clinical trials of basal compared to other insulins [4], [17]–[19]. Lower weight gain with basal insulin is believed to be a consequence of lesser hyperinsulinaemia in the post-prandial period, with consequent less hunger as a result of lower plasma glucose concentration, and perhaps concomitantly less frequent need for correction of hypoglycaemia with carbohydrates. We were not able to compare the rates of hypoglycaemic events between groups or in relation to HbA_1 c_ as primary care records only capture episodes known to the physician rather than events treated by family, paramedics or the patient themselves.

Insulin was often started when glycaemic control was very poor, with mean HbA_1c_ 9.9% (85 mmol/mol) (after exclusion of 8% with baseline HbA_1c_ <7.5% (58 mmol/mol)). This mean is higher than many clinical trials, which often have an upper as well as lower inclusion limit [4], [17], [19], demonstrating that trial populations may not always be representative of routine practice. It is consistent with the ad-hoc observational CREDIT study conducted in Europe, Canada and Japan [20]. Overall glucose lowering was good (means 1.0–1.7%) after 12 months and, unlike interventional studies, could not be biased by selection of patients or physicians. Nevertheless, because starting HbA_1c_ levels were high, control to target was not achieved in the majority of people even after one year.

This study has a number of limitations. While we were able to adjust for many potential confounding variables, severity of prevalent vascular disease (such as angina), prior long term glycaemic control, socioeconomic status and ethnicity were not routinely available on all patients in this observational dataset in the study years and recorded duration of diabetes has not been validated. While these variables are likely to be non-differential between the insulin types, we cannot exclude the presence of some residual confounding. Confounding by indication should be decreased as all subjects initiated insulin when they had poor glycaemic control, although different insulins may be started in different clinical circumstances such as ambulatory care versus in-patient treatment. We chose to include all-cause mortality rather than cardiovascular mortality because the recording of cause of death has been shown to be incomplete in general practice databases [21] so non-cardiovascular events could not be reliably excluded. HbA_1c_ may have been measured more frequently in those whose glycaemic control was poor resulting in higher mean levels during follow-up than were actually the case. As all patients should have levels measured after insulin introduction there should be no difference between insulin types. The finding that the number of patients who have a value measured remains relatively consistent over time, across treatment groups, is also reassuring. The first year of follow-up was excluded from all groups in the analysis of vascular events as the distribution of insulin use in terminally ill patients may not be even, causing bias, and any effect of the choice of therapy on our outcomes is likely to be in the medium- to long-term. The design was an intention-to-treat type analysis so we did not censor follow-up at the next change of glucose lowering treatment. As a result, the study did not investigate the association of individual insulins and cardiovascular outcomes but rather the mid-term consequences of one therapeutic decision when starting insulin. Later changes in treatment could be expected to relate to the efficacy and problems of the first insulin regimen.

Overall, while the number of patients lost due to inclusion/exclusion criteria appears high, the criteria for the initial data cut were untypically wide due to the two stage identification process (OGLD therapy and then insulin). For example, the requirement for a year of data recording pre- and post-insulin resulted in the removal of 29% of the total potential population but allowed time for recording of information on prevalent events as well as laboratory measurements. Many of those who had no baseline HbA1c also had less than one year of data pre-insulin. Many of the remaining exclusions occurred because the HbA_1c_ could not be interpreted rather than because it was not measured. The study covered a time of HbA_1c_ unit change in the UK.

In conclusion, in routine clinical practice no significant difference in the rate of incident macrovascular disease was found between first insulin type started during poor glycaemic control over a period of 1 to 3.6 years. The increased risk of microvascular disease between users of NPH and basal insulin was found in those who escalated from three but not two agents and so warrants further investigation. First time use of basal insulin was associated with a smaller increase in weight gain and a smaller decrease in HbA_1c_ in the first year compared to other insulins in some treatment groups. Glycaemic control was poor at baseline and, while mean HbA_1c_ decreased during insulin therapy, control remained poor across all groups.
